# Uterine carcinosarcomas: A case series of 9 cases from a low-income country

**DOI:** 10.1097/MD.0000000000039773

**Published:** 2024-10-04

**Authors:** Boubacar Efared, Halidou Hamadou Koura, Aïchatou Balaraba Abani Bako, Idrissa Boubacar, Habiba Salifou Boureima, Garba Mahamadou, Hassan Nouhou

**Affiliations:** a Faculté des Sciences de la Santé, Université Abdou Moumouni, Niamey, Niger; b Laboratoire d’anatomie et cytologie pathologiques, Hôpital National de Niamey, Niamey, Niger; c Laboratoire d’anatomie et cytologie pathologiques, Hôpital de Référence, Maradi, Niger; d Laboratoire d’anatomie et cytologie pathologiques, Hôpital Général de Référence, Niamey, Niger; e Service de gynécologie-obstétrique, Centre Hospitalier Régional Poudrière, Niamey, Niger.

**Keywords:** carcinosarcoma, pathology, prognosis, uterus

## Abstract

**Rationale:**

Uterine carcinosarcomas (UCS) are rare aggressive biphasic tumors classified as a subtype of high-grade uterine carcinomas. However, these tumors have particular histopathological features and clinical behavior with worse prognosis than high-grade uterine carcinomas.

**Patient concerns:**

The incidence of UCS is increasing and more studies are required to elucidate their clinical and histopathological characteristics. Herein, we report clinicopathological features of 9 cases of UCS in a low-income country.

**Diagnoses:**

We retrospectively collected all cases of UCS at our Pathology Department over a period of 4 years. The diagnosis was performed on formalin-fixed, paraffin-embedded and hematoxylin and eosin–stained surgical specimens.

**Interventions:**

Nine surgically treated cases of UCS have been registered, representing 12.67% of all uterine malignancies with a mean age of 58.88 years (range: 50–65 years). Abdominal pain and metrorrhagia were the main clinical presentations. The epithelial component of UCS was often a serous carcinoma (66.66%) and patients presented with large tumors (mean size of 9.24 cm, range of 5–19 cm), with advanced FIGO stages (stages III–IV) in 5/9 patients (55.55%).

**Outcomes:**

Follow-up data were available in 5/9 patients among which only 2 were alive 2 and 25 months after the surgical treatment (overall survival of 40%).

**Lessons:**

UCS are rare and aggressive uterine tumors with very poor prognosis especially in low-income countries.

## 1. Introduction

Uterine carcinosarcomas (UCS) are uncommon and aggressive biphasic tumors representing <5% of all uterine malignancies.^[1,2]^ These tumors show a mixture of carcinomatous component (usually a high grade endometrioid carcinoma, serous carcinoma or clear cell carcinoma) and a sarcomatous component (SC) that can be either homologous or heterologous.^[3,4]^ When the SC consists of mesenchymal tissues normally found in the uterus (leiomyosarcoma, endometrial stromal sarcoma) the tumor is considered as homologous, and heterologous when the differentiation of the SC shows extrinsic mesenchymal tissues (rhabdomyosarcoma, chondrosarcoma, liposarcoma).^[3]^ Previously, UCS have been considered as high grade uterine sarcomas and treated as such, but subsequent molecular studies have proved that these tumors are in fact high grade uterine carcinomas.^[4–6]^ Both carcinomatous and sarcomatous derived from a common precursor cell that undergoes divergent differentiation, mainly through a mechanisms known as epithelial-mesenchymal transition (EMT) or metaplasia during tumor’s evolution.^[1,4,7,8]^ A part from the EMT, some theories suggest that UCS may derive from other mechanisms^[4]^:

- The collision theory suggests that the epithelial carcinomatous component (EC) and the SC are 2 independent neoplasms.
- The conversion theory suggests that the SC derives from the EC during the evolution of the tumor.
- The composition theory suggests that the SC is a pseudosarcomatous stromal reaction to the presence of the EC.

UCS are aggressive tumors often diagnosed at advanced stages, their incidence is increasing and more studies are required to elucidate their clinical behavior, histopathological and molecular nature.^[9,10]^

Herein, our objective is to report clinicopathological features of 9 cases of UCS in a low-resource context with a literature review.

## 2. Methods

### 2.1. Patients selection

This is a retrospective study including all cases of UCS diagnosed at our pathology department from February 2020 to March 2024 (a period of about 4 years). Clinical and follow-up data were retrieved from pathology requests forms and telephone calls of patients or their relatives and treating physicians.

Cases of other uterine cancers (pure carcinomas, pure sarcomas, malignant trophoblastic diseases or malignant mixed tumors showing benign epithelial or benign mesenchymal components) have been excluded from the study.

### 2.2. Histopathological diagnosis

The archival tissue blocks of all cases have been reviewed and additional cuts were performed to assess the nature (whenever possible), the proportion of the sarcomatous and carcinomatous components and the presence of lymphovascular invasion (LVI) in each case.

The histopathological diagnosis was made on formalin-fixed, paraffin-embedded tissues stained by hematoxylin and eosin (H&E).

The tumor staging was performed according to the FIGO (Fédération Internationale de Gynécologie et d’Obstétrique) staging system.^[11]^

Publication of this case series was approved by the Institutional Board Review of Hôpital National de Niamey (Reference: No. 0248/DGAHNN/DAF/SGRH).

Written consent was obtained from the patients for the purpose of publication of cases details and images.

## 3. Results

### 3.1. Clinical features

The Table 1 summarizes the clinical characteristics of our series.

**Table 1:** Clinical features or our series of uterine carcinosarcoma (UCS).

Cases	Age	Symptoms	Tumor sites	Surgery	Follow-up
1	50	Abdominal pain, ascite	Omentum, uterus	TH + BSO + Om + Ap.	Lost to follow-up
2	57	Abdominal pain, vaginal bleeding	Uterus	TH + BSO + Om	Alive (25 months after surgery)
3	62	Abdominal pain, vaginal bleeding	Uterus, cervix	TH + BSO	Died (1 week after surgery)
4	55	Abdominal pain, vaginal bleeding	Uterus	TH + BSO	Lost to follow-up
5	64	Abdominal pain, vaginal bleeding, ascite	Uterus, Omentum, lymph nodes	TH + BSO + Om + Lymph.	Died (14 months after uterine biopsy)
6	65	Abdominal pain, vaginal bleeding, ascite	Omentum, uterus	TH + BSO + Om	Lost to follow-up
7	65	Abdominal pain, vaginal bleeding, ascite	Omentum, ovaries, uterus	TH + BSO + Om + Ap.	Lost to follow-up
8	60	Abdominal pain, vaginal bleeding	Uterus	TH + BSO	Died (3 weeks after surgery)
9	52	Abdominal pain, vaginal bleeding	Uterus, cervix	TH + BSO	Alive (2 months after surgery)

Ap = appendicectomy, BSO = bilateral salpingo-oophorectomy, HT = total hysterectomy, Lymp = lymphadenectomy, Om = omentectomy.

During our study-period of about 4 years (2020–2024), 71 cases of malignant uterine tumors have been registered among which 9 cases of UCS (12.67% of uterine malignancies). All patients were postmenopausal women with a mean age of 58.88 years (range of 50–65 years). All patients presented with abdominal pain associated with vaginal bleeding, whereas 4/9 patients (44.44%) had associated ascite. Total hysterectomy and bilateral salpingo-oophorectomy (TH + BSO) was performed in all patients, omentectomy was also associated in 5 patients (55.55%), appendicectomy in 2 cases (22.22%), and pelvic lymphadenectomy in 1 patient who was the unique case to had histopathological preoperative diagnosis from a uterine curettage (case 5). The tumor was located in the uterine corpus in all cases, extended to the uterine cervix in 2 cases (cases 3 and 9), to the omentum in 4 cases (44.44%). The case 5 was the only patient where lymphadenectomy has been performed and it showed lymph node metastasis. The case 7 had also bilateral ovarian metastases.

Our follow-up data were poor. Many cases (4/9 patients) have been lost to follow-up despite several attempts to contact their relatives or treating physicians. Among the remaining cases, 2 patients (25%) died shortly after the surgical treatment (cases 3 and 8) after 1 and 3 weeks respectively. These patients did not receive any adjuvant treatment as they died before the pathological diagnosis was made. Also, 1 patient died (case 5) 14 months after the diagnoses (biopsy); she was treated by chemotherapy + radiotherapy after the surgical treatment. The patient 2 (case 2) was alive 25 months after the surgical treatment, but now lost to follow-up; she was also treated by chemotherapy. Patient 9 was also alive 2 months after the surgical treatment.

### 3.2. Pathological features

Pathological features of our series are presented in Table 2.

**Table 2:** Pathological features of our series of uterine carcinosarcoma (UCS).

Cases	Macroscopic feature/size	Microscopy		Metastasis		Lymphovascular invasion	FIGO stage
		EC (proportion)	SC	EC	SC		
1	Solidocystic/6 cm	Serous carcinoma (25%)	Spindle cell sarcoma	+	+	−	IVB
2	Solid/10 cm	Clear cell carcinoma (40%)	Spindle cell sarcoma	−	−	+	IA
3	Solid/5 cm	Endometrioid carcinoma (20%)	Rhabdomyosarcoma	−	−	+	II
4	Polypoid/6.5 cm	Endometrioid carcinoma (30%)	Spindle cell sarcoma	−	−	+	IB
5	Solid/6.7 cm	Serous carcinoma (30%)	Spindle cell sarcoma	+	+	+	IVB
6	Polypoid/5 cm	Serous carcinoma (60%)	Spindle cell sarcoma	+	+	−	IVB
7	Solid/17 cm	Serous carcinoma (25%)	Rhabdomyosarcoma	+	−	−	IVB
8	Solid/8 cm	Serous carcinoma (40%)	Chondrosarcoma	−	−	−	IA
9	Solid/19 cm	Serous carcinoma (10%)	Rhabdomyosarcoma	−	−	+	IIIA

EC = epithelial component, FIGO = Fédération Internationale de Gynécologie et d’Obstétrique, SC = sarcomatous component.

**Figure 1.**
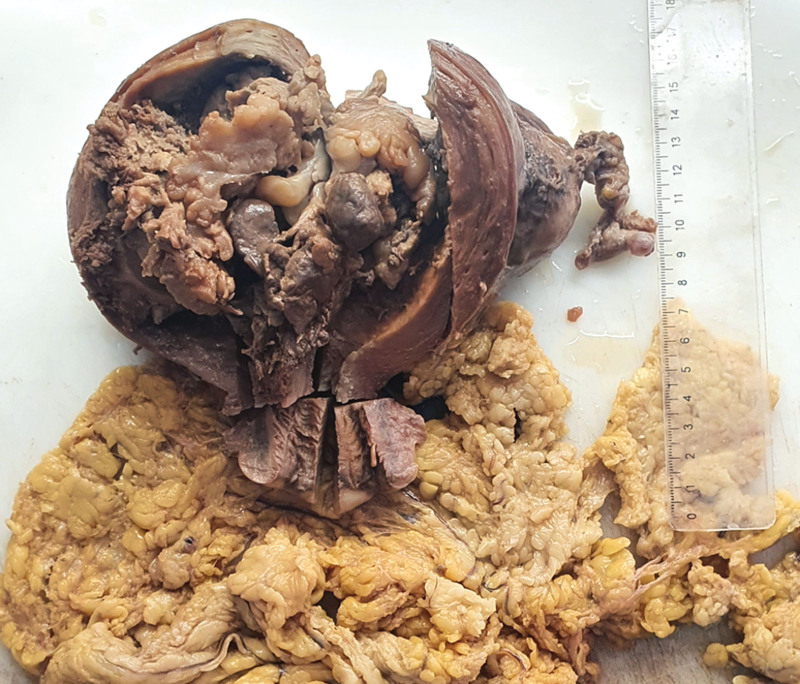
The macroscopic view of a patient with UCS (case 2) showing a solid tumor filling the uterine cavity. The resected omentum is not infiltrated by the tumor.

The macroscopic features of the resected tumors were almost solid masses filling the uterine cavity (Fig. 1) with a mean tumor size of 9.24 cm (range of 5–19 cm).

**Figure 2.**
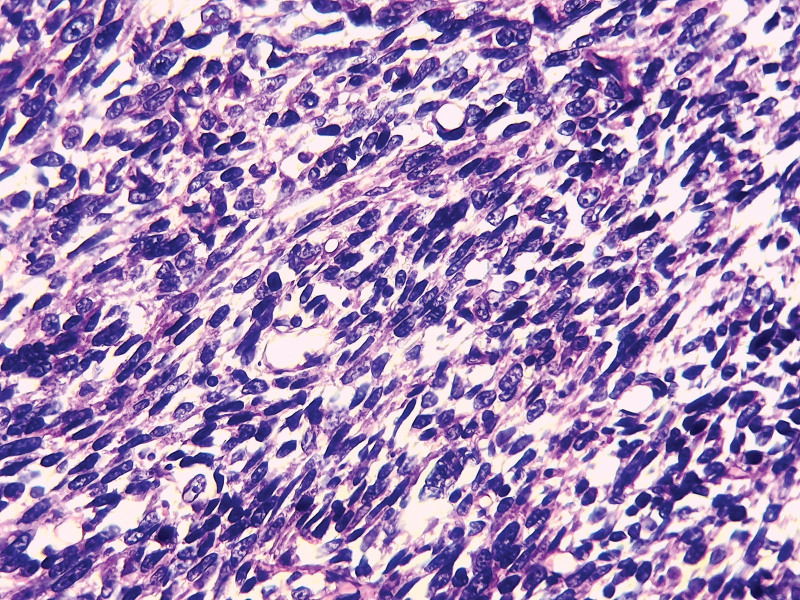
The histopathological images of UCS showing a sarcomatous component (SC) with spindle cells (case 1); (H&E ×400).

**Figure 3.**
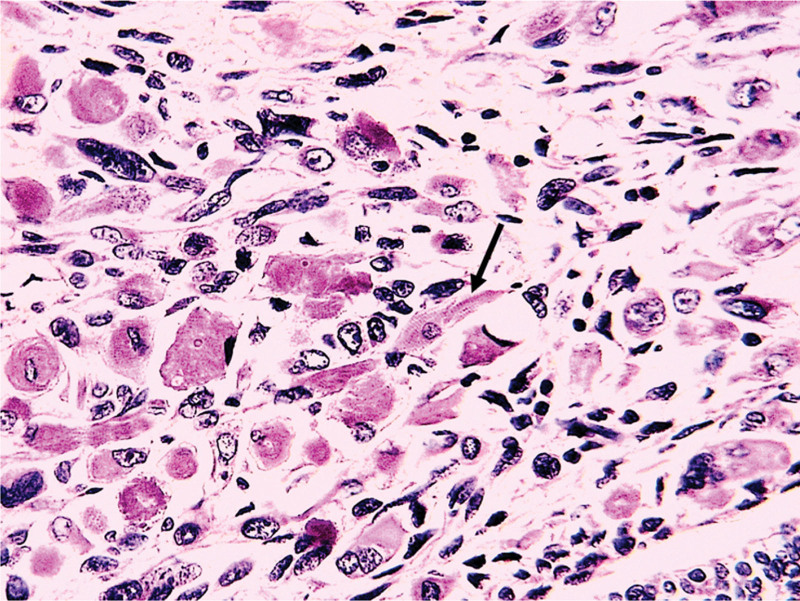
The histopathological view of UCS with rhabdomyomatous differentiation (Black arrow) (case 3); (H&E ×400).

**Figure 4.**
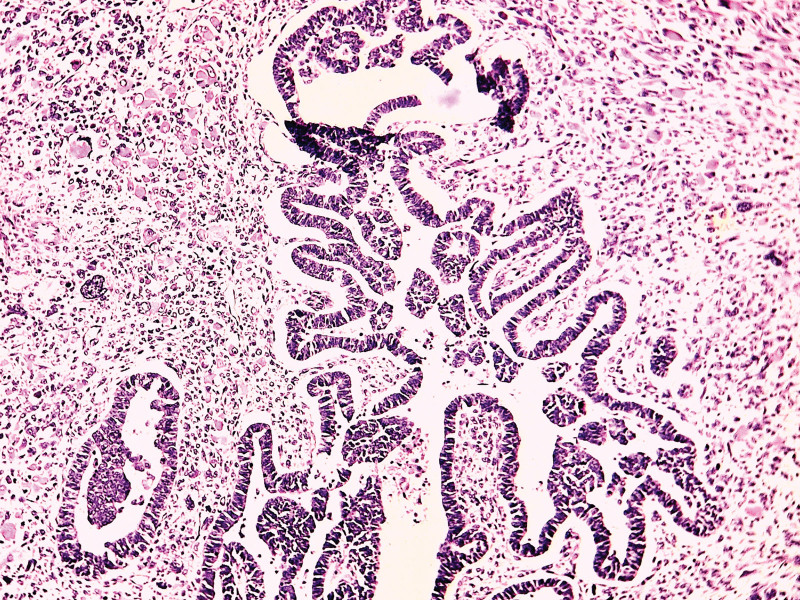
The carcinomatous components of UCS showing a serous carcinoma (case 7); (H&E ×200).

**Figure 5.**
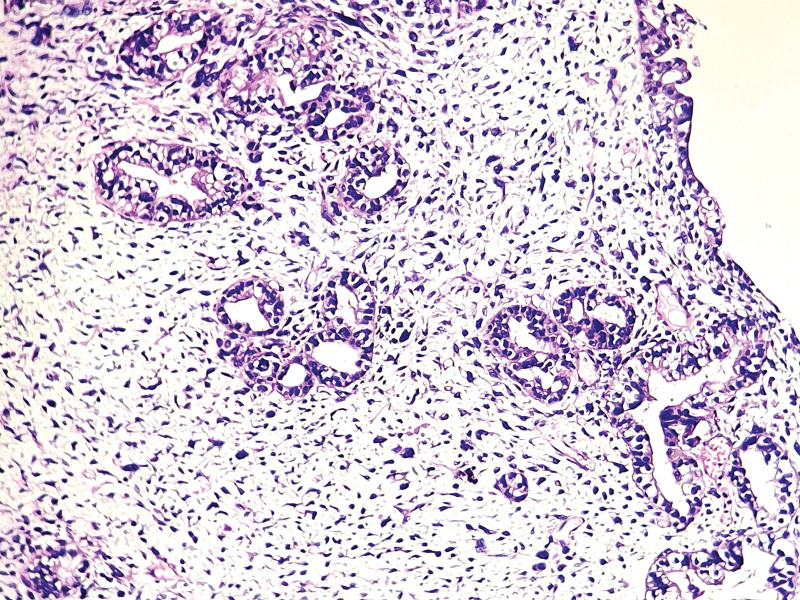
The carcinomatous components of UCS with clear cell carcinoma (case 2). (H&E ×200).

On histological analysis based on morphology only without immunohistochemistry (unavailable in our country), all cases of UCS presented mixed, intricated epithelial and SCs with most cases showing predominant SC (8/9 cases) with rhabdomyomatous differentiation in 3 cases (33.33%) (Fig. 2), chondromatous differentiation in 1 case (11.11%) and spindle cell differentiation in 5 cases (55.55%) (Fig. 3). The carcinomatous component is only predominant in 1 case (case 6), ranging from 10% to 60% of the tumor surface. This component is essentially serous carcinomas (6 cases, 66.66%) (Fig. 4), high grade endometrioid carcinomas (2/9 cases) and clear cell carcinoma (1/9 case) (Fig. 5).

Four patients had omental metastases associated with lymph nodes (case 5) and ovarian (case 7) secondary locations. All the omental and lymph nodes sites showed biphasic tumors, however the ovarian metastases were only made of carcinomatous components. Lymphovascular invasion (LVI) was detected in 5 patients (55.55%). Five patients (50%) presented with advanced tumor stages (FIGO stages III–IV).

## 4. Discussion

Uterine carcinosarcomas (UCS) are a rare and aggressive subtype of uterine high grade carcinomas.^[3,4]^ Their incidence is increasing in certain countries like USA, and these tumors represent <5% of all uterine malignancies and occur in postmenopausal women.^[1,4,9]^ We found a high prevalence of UCS in our study, representing 12.67% of all uterine malignancies, with a mean age of 58.88 years. All patients were postmenopausal women but they were younger compared to previous reports in the literature where the mean or median age of patients was above 65 years.^[8,12,13]^ The younger age of our patients could be due to a small sample or perhaps due to the African context of our study (all patients were Black), as studies have reported increased incidence of UCS in Black women.^[9]^ Obesity or increased estrogen exposition (intrinsic or extrinsic) are the main risk factors of uterine carcinomas, and some studies have reported increased risk of developing UCS in patients with breast cancer previously treated by estrogen receptor modulators in patients with.^[14,15]^ None of our patients had breast cancer or any previous treatment by hormonal therapy. The clinical presentation of our cases was typical of uterine malignant tumors: abdominal pain, vaginal bleeding with or without ascite when the tumor invaded intra-abdominal structures.^[13]^ Abdominal pain and vaginal bleeding were associated with ascite in 4 of our patients (44.44%) which had advanced stage tumors with abdominal extension.

The definitive diagnosis of UCS relies on pathological analysis of the uterine curettage (biopsy) or the hysterectomy specimens. The histological diagnosis of UCS is straightforward by the standard pathological technique (formalin-fixed, paraffin-embedded tissues stained by H&E); the immunohistochemistry (IHC) could be useful in order to precise certain sarcomatous differentiations when they are not obvious (especially spindle cell sarcomas). In our study, we did not use IHC for the diagnosis of UCS as this special technique is not available in the entire country, also our pathology laboratory is the unique functional public pathology laboratory in the country at the time of this study, fortunately another pathology laboratory has just opened at a public hospital recently. Our patients presented with large solid tumors usually filling the entire uterine cavity, with a mean size of 9.24 cm (range of 5–19 cm). This reflects the late diagnosis in our context because of poor medical conditions, although some studies showed that tumor size had no consistent prognostic value.^[12,13]^ We have found that the SC is often predominant over the EC that is often a serous carcinoma (6/9 cases) followed by high grade endometrioid carcinoma (2/9 cases). These histological features are commonly reported in the literature as the EC of UCS is a high grade uterine carcinoma with often a serous or endometrioid differentiation.^[2,3,8]^ The SC in our series is a spindle cell sarcoma without any obvious differentiation in 5/9 cases. Without IHC we cannot speculate either these sarcomas were leiomyosarcoma, rhabdomyosarcoma, fibrosarcomas or other spindle cell sarcomas. Only 4 cases showed obvious sarcomatous differentiation (3 cases of rhabdomyosarcoma and 1 case of chondrosarcoma). In the literature, there were contradicting reports regarding the prognostic value of the sarcomatous differentiation in UCS (heterologous or homologous).^[8,12,13]^ Most previous studies on UCS did not find significant prognostic value of the nature of the mesenchymal component (homologous or heterologous) on survival.^[8,13]^ However, some authors have found that the presence of a homologous component in the SC was associated with a more favorable outcome compared to tumors with heterologous SC.^[12,16]^ In our study, the 2 deceased patients (2 cases out of 3) had heterologous component (rhabdomyosarcoma and chondrosarcoma), the remaining patient had spindle cell sarcoma as a SC component, without IHC we could not determine if this was homologous or heterologous component. The FIGO stage remains the most robust prognostic survival factors in patients with UCS, and advanced stages (FIGO stages III and IV) are usually associated with a higher risk of death.^[4,13]^

In our study, 5/9 patients (55.55%) presented with advanced FIGO stages (stages III–IV) with tumor extension to the uterine serosa, omentum, ovaries or lymph nodes. In all but 1 (patient 7) the extrauterine tumor locations showed biphasic differentiation. The case 7 showed only carcinomatous component in the omentum and ovaries.

All cases have been treated by surgery (mainly total hysterectomy + bilateral salpingo-oophorectomy) and follow-up data were available only in 5 patients (55.55%) among which only 2 patient were known alive 2 months (patient 9) and 25 months (patient 2) after surgery. However patient 2 has been lost to follow-up after 25 months. Two patients died shortly after surgery (1 and 3 weeks) before the histopathological diagnosis was disclosed (in our pathology laboratory, the mean time to deliver the diagnosis is about 1 to 2 months). We could speculate that all these lost to follow-up cases had died as the aggressiveness of the disease could not allow survival without adjuvant therapy. A part from the biological aggressiveness of UCS, we think that poor medical infrastructures (delayed diagnosis, poor medical follow-up) are to blame for this dismal prognosis. The UCS have worse prognosis than other high grade uterine carcinomas and the 5-year overall survival is <20%.^[10,13]^

Despite some limitations (small sample size, short study-period, retrospective and monocentric study), our study confirmed the commonly established facts about UCS: rare and aggressive uterine tumors with very poor prognosis especially in low-income countries with difficult medical conditions.

## 5. Conclusion

Uterine carcinosarcomas are uncommon biphasic tumors considered as a subtype of uterine high grade carcinomas of postmenopausal women presenting usually with abdominal pain and metrorrhagia. Patients with UCS are often diagnosed at advanced stages with a high risk of mortality especially in low-income areas with poor medical conditions.

## Author contributions

**Conceptualization:** Boubacar Efared.

**Data curation:** Boubacar Efared, Halidou Hamadou Koura, Aïchatou Balaraba Abani Bako, Idrissa Boubacar, Habiba Salifou Boureima, Garba Mahamadou.

**Supervision:** Hassan Nouhou.

**Writing – original draft:** Boubacar Efared.

**Writing – review & editing:** Halidou Hamadou Koura, Aïchatou Balaraba Abani Bako, Idrissa Boubacar, Habiba Salifou Boureima, Garba Mahamadou, Hassan Nouhou.
